# Unveiling the role of IGF1R in autism spectrum disorder: a multi-omics approach to decipher common pathogenic mechanisms in the IGF signaling pathway

**DOI:** 10.3389/fgene.2024.1483574

**Published:** 2024-09-23

**Authors:** Kang Yang, Tian Zhang, Ruize Niu, Liyang Zhao, Zhonghe Cheng, Jun Li, Lifang Wang

**Affiliations:** ^1^ National Clinical Research Center for Mental Disorders, Peking University Sixth Hospital, Beijing, China; ^2^ Affiliated Mental Health Center & Hangzhou Seventh People’s Hospital and School of Brain Science and Brain Medicine, Zhejiang University School of Medicine, Hangzhou, China; ^3^ Affiliated Mental Health Center of Kuming Medical University, Yunnan Psychiatric Hospital, Kunming, China

**Keywords:** autism spectrum disorder, IGF signaling pathway, multi-omics, IGF1R, neurodevelopmental mechanisms

## Abstract

Autism spectrum disorder (ASD) is a complex neurodevelopmental condition marked by impairments in social interaction, communication, and repetitive behaviors. Emerging evidence suggests that the insulin-like growth factor (IGF) signaling pathway plays a critical role in ASD pathogenesis; however, the precise pathogenic mechanisms remain elusive. This study utilizes multi-omics approaches to investigate the pathogenic mechanisms of ASD susceptibility genes within the IGF pathway. Whole-exome sequencing (WES) revealed a significant enrichment of rare variants in key IGF signaling components, particularly the IGF receptor 1 (IGF1R), in a cohort of Chinese Han individuals diagnosed with ASD, as well as in ASD patients from the SFARI SPARK WES database. Subsequent single-cell RNA sequencing (scRNA-seq) of cortical tissues from children with ASD demonstrated elevated expression of IGF receptors in parvalbumin (PV) interneurons, suggesting a substantial impact on their development. Notably, IGF1R appears to mediate the effects of IGF2R on these neurons. Additionally, transcriptomic analysis of brain organoids derived from ASD patients indicated a significant association between IGF1R and ASD. Protein-protein interaction (PPI) and gene regulatory network (GRN) analyses further identified ASD susceptibility genes that interact with and regulate IGF1R expression. In conclusion, IGF1R emerges as a central node within the IGF signaling pathway, representing a potential common pathogenic mechanism and therapeutic target for ASD. These findings highlight the need for further investigation into the modulation of this pathway as a strategy for ASD intervention.

## 1 Introduction

Autism spectrum disorder (ASD) is a complex neurodevelopmental condition characterized by deficits in social interaction, communication, and repetitive behaviors. It is significantly influenced by genetic factors, yet the genetic heterogeneity associated with ASD is remarkably high (Ghafouri-Fard et al., 2023). In recent years, numerous ASD susceptibility genes have been identified and validated, but the pathogenic pathways through which these genes exert their effects remain diverse and lack a unified pathological mechanism.

The IGF signaling pathway is essential for regulating cell growth, development, and metabolism (Mancarella et al., 2021). This system includes two ligands, IGF1 and IGF2, and their respective receptors, IGF1R and IGF2R (Forbes et al., 2020). Various IGF Binding Proteins (IGFBPs) and IGFBP-related proteins modulate the extracellular functions and activities of IGF1 and IGF2 (Li et al., 2020; Liu et al., 2023; Stuard et al., 2020). IGF1 and IGF2 primarily act through IGF1R, activating downstream PI3K/AKT and MAPK pathways, which regulate cell proliferation, differentiation, survival, and metabolism (Takahashi, 2019). The PI3K/AKT pathway, involving Phosphoinositide 3-kinase (PI3K) and Protein Kinase B (AKT), promotes cell growth and survival and regulates protein synthesis and metabolism via the mammalian target of rapamycin (mTOR) (Gupta and Jansson, 2019). The MAPK pathway, involving Ras, RAF, MEK, and ERK, influences gene expression and cell proliferation and differentiation (Devis-Jauregui et al., 2021). IGF2, upon binding to IGF2R, is internalized and degraded by lysosomes, thereby regulating extracellular IGF2 levels (Beletskiy et al., 2021; Alberini, 2023). Proper regulation of the IGF signaling pathway is crucial for maintaining cellular growth, function, and metabolic balance, and its dysregulation is linked to diseases such as cancer, diabetes, and growth disorders (Mancarella et al., 2021; Forbes et al., 2020; Altieri et al., 2019; Okuyama et al., 2021; Stefani et al., 2021; Jiating et al., 2019; Yang et al., 2020; Chaumont-Dubel et al., 2020). Recent studies have suggested that the use of IGF1 analogs or IGF2 can ameliorate social and behavioral deficits in individuals with ASD (Neul et al., 2022; Pizzarelli et al., 2023), implicating the insufficient IGF signaling in the pathogenesis of ASD. However, the pathogenic mechanisms remain underexplored.

**FIGURE 1:**
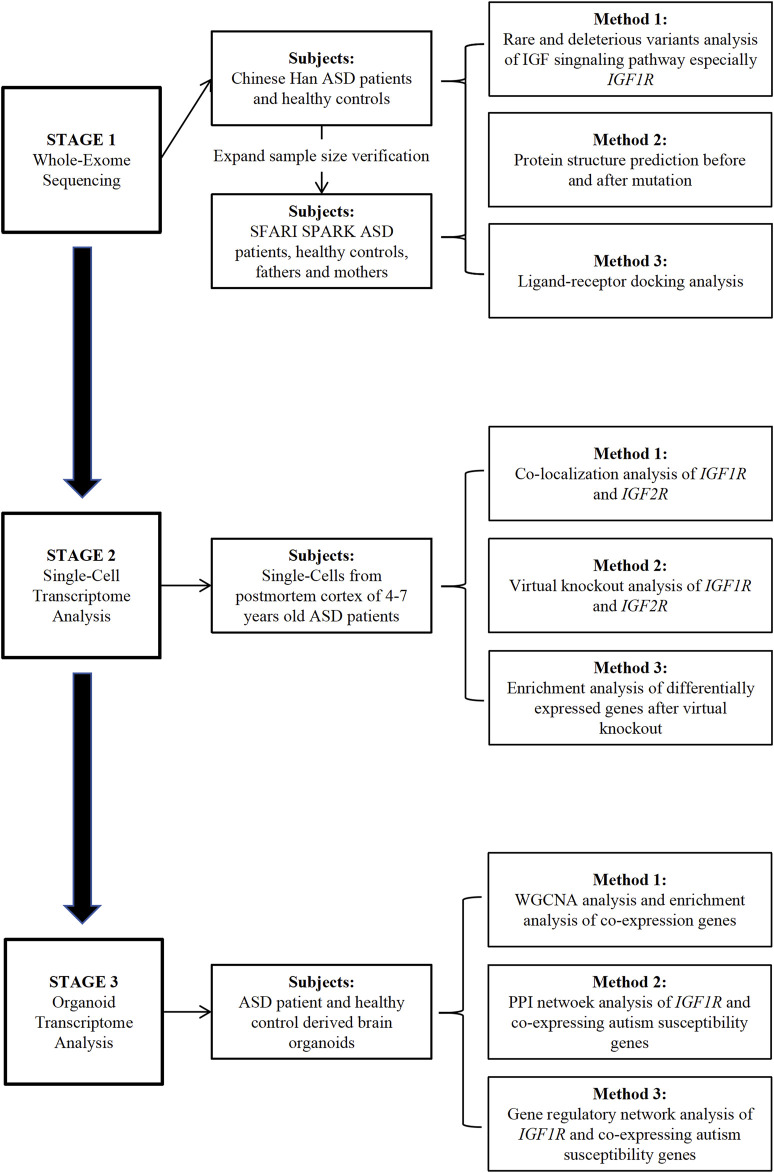
Study Workflow and Design. The diagram depicts the study workflow encompassing whole-exome sequencing, single-cell transcriptome analysis, and transcriptome analysis of autism organoids.

In this research, we adopted an extensive multi-omics strategy to uncover potential shared pathogenic mechanisms associated with ASD susceptibility genes, focusing on key molecules within the IGF signaling pathway. Initially, whole-exome sequencing (WES) revealed a notable enrichment of rare variants in essential IGF signaling components, particularly IGF receptor 1 (IGF1R), in a cohort of Chinese Han individuals diagnosed with ASD, as well as in ASD patients from the SFARI SPARK WES database. Following this, single-cell RNA sequencing (scRNA-seq) of cortical tissues from children with ASD showed a marked expression of IGF-Rs in parvalbumin (PV) interneurons, indicating a significant influence on their development. Interestingly, the effect of IGF2R on these neurons might be mediated through IGF1R. Lastly, transcriptomic analysis of brain organoids derived from ASD patients highlighted a significant link between IGF1R and ASD. Protein-protein interaction (PPI) and gene regulatory network (GRN) analyses further pinpointed ASD susceptibility genes that interact with and regulate IGF1R expression (Figure 1).

The impetus for this study arises from the pressing need to identify and validate a unified pathological mechanism of ASD and to pinpoint molecular targets within the IGF signaling pathway that could serve as potential therapeutic interventions. The identification of IGF1R as a central node suggests a potential convergent pathological pathway for the diverse genetic factors associated with ASD, providing new insights into its molecular underpinnings. By unraveling the complex network of interactions involving IGF1R and its regulatory mechanisms, we aim to deepen our understanding of ASD pathogenesis and lay the groundwork for novel therapeutic strategies. These findings underscore the necessity for further exploration of the precise molecular interactions and regulatory networks involved. Future research employing induced pluripotent stem cell (iPSC) technology to generate brain organoids, alongside gene editing techniques in animal models to manipulate *IGF1R* and related genes, holds promise for elucidating the mechanisms of the IGF signaling pathway in the pathogenesis of ASD.

## 2 Materials and methods

### 2.1 Ethics statement

This study was approved by the Ethics Committee of Peking University Sixth Hospital, China. All participants provided written informed consent to participate in this study. The informed consents of children were obtained from their legal guardians.

### 2.2 Participants

All the participants were of Chinese Han ancestry and recruited at Peking University Sixth Hospital, China.

The diagnosis of autism was established by two senior psychiatrists. Autistic patients met the criteria of the Diagnostic and Statistical Manual of Mental Disorders, fourth edition (DSM-Ⅳ) for autism. Additional criteria for patient inclusion were Autism Behavior Checklist score ≥53 and Childhood Autism Rating Scale ≥35. The exclusion criteria were as follows: diagnosed with Asperger syndrome, Rett syndrome, pervasive development disorder not otherwise specified, fragile X syndrome, tuberous sclerosis, a previously identified chromosomal abnormality, dysmorphic features, or any other neurological conditions. For healthy control, only adults (older than 18 years old) and individuals did not be diagnosed with familial inherited diseases or psychiatric disorders were included.

In this study, a total of 786 participants were recruited. Firstly, we recruit 197 children with autism and 299 healthy controls. And then, we included 71 autism families, in which at least one child was diagnosed with ASD and neither parent was diagnosed with ASD. These families were unrelated to the aforementioned samples. The family cohort consisted of 89 probands with ASD, 45 healthy siblings, 7 siblings with other disorders, 142 parents, 6 grandparents, and 1 uncle, totaling 290 individuals. Statistical analysis revealed no significant difference in the distribution of male and female participants between the autistic group (197 children with autism and 89 autistic probands) and the healthy control group (χ^2^ = 0.93, *P* = 0.334).

### 2.3 Whole exome sequencing and sample quality control

Exome capture was performed using Agilent SureSelect HumanAll Exon V6 kit (Agilent Technologies, Santa Clara, CA) following the manufacturer’s recommended protocol. Paired-end sequencing (2 × 150 basepair) was performed on an Illumina (San Diego, CA) NovaSeq 6000. Whole exome sequencing was obtained on a total of 786 individuals.

During the sample quality control process, sex verification was conducted by comparing the sequencing depth of X chromosome and autosomal variant sites. Samples with sex discrepancies were further confirmed by PCR and subsequently excluded. Pairwise concordance analysis using SNV data was performed to identify and exclude samples from the control group with abnormal kinship, duplicate samples, or samples with known relationship discrepancies. Consequently, 17 samples were excluded, resulting in a final cohort of 769 samples, comprising 196 children with autism, 294 healthy controls, and 69 autism families. The autism families included 87 probands with ASD, 40 healthy siblings, 7 siblings with other disorders, 138 parents, 6 grandparents, and 1 uncle. Whole-exome sequencing data from a total of 263 ASD patients (196 children with autism and 67 probands with ASD, with one proband randomly selected from each set of related probands to avoid redundancy) and 294 healthy controls were utilized for subsequent analysis focusing on the IGF signaling pathway.

Pre-processed whole exome sequencing data were obtained from the Simons Foundation Powering Autism Research (SFARI SPARK) initiative (SPARK Consortium, 2018). The dataset comprises 44,304 individuals diagnosed with ASD, 14,368 unaffected siblings, 17,332 fathers, and 29,724 mothers.

### 2.4 Sequencing data bioinformatics analysis

The raw data was filtered by fastp-0.20.0 (Chen et al., 2018) to trim the adapter sequences. Subsequently, we used Sentieon-202010 (Kumar et al., 2022) and BWA-0.7.17-r1188 (Li and Durbin, 2009) to align the clean sequence reads to the human reference genome (GRCh37). Then, we used Sentieon to process the quality control of the mapped data and further call the mutations. Variant call accuracy was estimated using the Sentieon Variant Quality Score Recalibration (VQSR) approach. Variants that failed VQSR were filtered. Only sequencing data of depth > 20 was included in further analysis. Sample quality control was according to the similarity between the filtered variants of each sample and the consistence of the imputed sex and the recording sex. The final mutation calls was annotated by ANNOVAR (Wang et al., 2010), which included gene annotation, aminoacid change annotation, dbSNP identifiers and damaging prediction. Brain expression annotation for each variants was conducted using the pext data. In addition, we use Prot2HG to do protein domain annotation.

### 2.5 Define rare or deleterious variants

Variants were defined as rare if their allele frequency is less than 0.01 in the control cohort and one of the public database (1000g_ALL, 1000g_EAS, esp6500siv2_all, ExAC_ALL, gnomad_controls, and gnomad_controls_EAS (Devuyst, 2015; Karczewski et al., 2017)).

Twelve tools (SIFT, Polyphen2_HDIV, Polyphen2_HVAR, LRT, MutationTaster, MutationAssessor, FATHMM, PROVEAN, fathmm-MKL_coding, MetaSVM, MetaLR and CADD (Adzhubei et al., 2013; Choi and Chan, 2015; Ioannidis et al., 2016; Schubach et al., 2024; Schwarz et al., 2014; Stanek et al., 2020; Vaser et al., 2016; Reva et al., 2011; Garcia et al., 2022; Chen et al., 2024)) were used to do computational prediction of deleteriousness for each variant. The level of deleteriousness was divided into deleterious (D), probably deleterious (P) and tolerable (T) according to each tool’s criteria (CADD: 15 threshold for deleterious and tolerable). The number of twelve prediction results in each deleterious level (deleterious, probably deleterious and tolerable) were counted as nD, nP and nT, respectively. The definition of the HARM value is given by the equation: HARM = nD + nP × 0.4 − nT. Two criteria to estimate the deleteriousness of variants was used in this study: ① nD > 0; ② HARM > 0.

### 2.6 Genetic mutation burden and association analysis

We evaluated the mutation burden for each individual within the autism and control cohorts. Depending on the normality of the data distribution and the homogeneity of variances between the two groups, we employed different statistical tests for one-tailed significance testing: T-test (normal distribution, equal variances), Welch’s T-test (normal distribution, unequal variances), or Mann-Whitney U test (non-normal distribution). Association analyses were performed at both the variant and gene levels. For variant-level analysis, we enumerated the number of individuals carrying each variant and those not carrying the variant in both cases and controls. One-tailed Fisher’s exact test was applied to identify variants significantly enriched in the autism cases. For gene-level analysis, we tallied the specific types of variants within each gene in both cases and controls. Subsequently, one-tailed Fisher’s exact test was utilized to detect genes significantly enriched with impactful variants in autism cases. To correct for multiple testing, we applied the Benjamini-Hochberg correction and Bonferroni correction to the *p*-values.

In this study, only variants with call rates exceeding 0.95 were included in the analyses. In the association analysis, adjustments for covariates such as age and gender were not performed, as the genotype remains unaffected by variations in age, gender, or inheritance probability.

### 2.7 Gene enrichment analysis

In postmortem brain samples from 4-7-year-old autism patients, differentially expressed genes in PV interneurons (PV InN) were identified through virtual knockout (KO) analysis. Additionally, in brain organoids, genes related to the IGF signaling pathway and autism susceptibility, which are part of co-expressed gene modules associated with autism, were also examined. To explore whether these genes were enriched in any known gene sets, such as those from gene ontology (GO) and KEGG pathways, we utilized Metascape (http://metascape.org/gp/index.html#/main/step1) for pathway enrichment analysis (Zhou et al., 2019).

### 2.8 Protein stucture and domin prediction and ligand-receptor docking analysis

The predicted structures of IGF1R, IGF1 and IGF2 were generated by Alphafold (Jumper et al., 2021), and the predicted domains of IGF1R were generated by SMART (Letunic et al., 2021). To ensure the accuracy of the docking results, the protein was prepared by the AutoDockTools-1.5.7 (Morris et al., 2008), and the water molecules were manually eliminated from the protein and the polar hydrogen was added. Docking Web Server (GRAMM) was used for protein-protein docking (Katchalski-Katzir et al., 1992; Vakser, 1996). The resulting protein-protein complex was also manually optimized by removing water and adding polar hydrogen by the AutoDockTools-1.5.7. Finally, the protein-protein interactions were predicted and the protein-protein interaction figure was generated by ChimeraX (Meng et al., 2023).

### 2.9 Protein-protein interaction network analysis

STRING (https://string-db.org/) was used to do protein network analysis to get the relationship of the given protein set (Szklarczyk et al., 2023).

### 2.10 Virtual knockout analysis

To elucidate the effect of gene KO on specific cell type function, we extract the snRNA-seq data of specific cell type and used the expression matrix of genes × cells as the input for scTenifoldKnk (Osorio et al., 2022). Briefly, we obtained the pre-processed snRNA-seq data, generated by Velmeshev et al. (The pre-processed details were available at https://autism.cells.ucsc.edu) (Velmeshev et al., 2019) and used the expression matrix of 36,501 genes × 72 PV InN cells from the sample across 4–7 years old as the input for scTenifoldKnk. We constructed the single-cell gene regulatory network (scGRN) and then knocked out *IGF1R or IGF2R*. The virtual KO perturbed genes with FDR-corrected *P* < 0.05 were selected as differentially expressed.

### 2.11 Transcriptomic analysis of autistic patients’ brain-like organoids

The pre-processed transcriptomic data of autistic patients' brain-like organoids were obtained from the GEO public database under the accession number GSE61476 (The pre-processed details were available at https://www.ncbi.nlm.nih.gov/pmc/articles/PMC4519016/) (Mariani et al., 2015). The sample sizes were as follows: day 0 of *in vitro* culture (autism group n = 2, control group n = 3), day 11 (autism group n = 8, control group n = 11), and day 31 (autism group n = 8, control group n = 13). Differential expression analysis of RNA-Seq data was conducted using the DESeq2 package in R (Costa-Silva et al., 2017). Principal component analysis (PCA) for dimensionality reduction and data visualization was performed using the FactoMineR and factoextra packages (Seredin et al., 2024). Weighted gene co-expression network analysis (WGCNA) was carried out using the WGCNA package to identify gene co-expression modules (Langfelder and Horvath, 2008), and a gene regulatory network was constructed using the GENIE3 package (Huynh-Thu and Geurts, 2018). Data visualization and result interpretation were done using packages such as ggplot2 and ComplexHeatmap (Gu et al., 2016).

## 3 Results

### 3.1 Analysis of rare variants in key factors of the IGF signaling pathway

**FIGURE 2:**
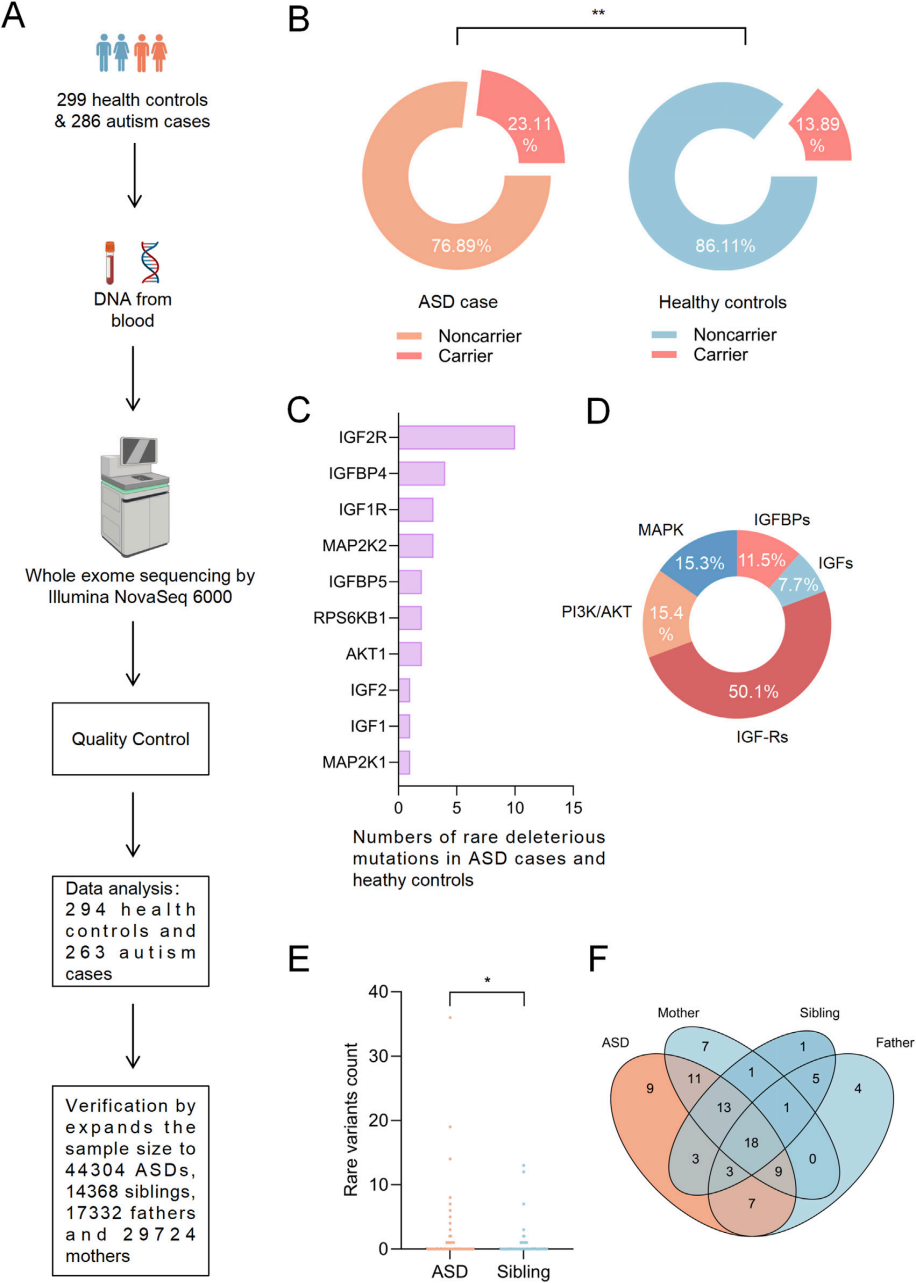
Key Findings on Rare Variants in the IGF Signaling Pathway in Han Chinese Autism Cohorts and SFARI SPARK Database. **(A)** Whole-exome sequencing workflow for Han Chinese ASD patients and healthy controls. The procedure involves DNA collection from 299 healthy controls and 286 autism cases, followed by sequencing using the Illumina NovaSeq 6000 platform. Quality control measures and subsequent data analysis were performed. Additionally, the sample size was expanded for validation using public data from the SFARI SPARK WES dataset, comprising 44,304 ASD cases, 14,368 siblings, 17,332 fathers, and 29,724 mothers. **(B)** Comparison of rare variant carriers in the IGF signaling pathway between autistic patients and healthy controls. **(C)** Distribution of 26 rare deleterious variants in 10 key IGF signaling pathway molecules among Chinese Han individuals with autism and healthy controls. **(D)** Functional classification of key IGF signaling molecules: IGFBPs (11.5%), IGFs (7.7%), IGF-Rs (50%), PI3K/AKT factors (15.4%), and MAPK factors (15.3%). **(E)** Further analysis of rare variants in *IGF1R* using expanded sample size from the SFARI SPARK WES public dataset, including 44,304 ASD cases and 14,368 siblings. **(F)** Further screening of rare deleterious variants in *IGF1R* exclusively present in ASD patients, utilizing an expanded sample size from the SFARI SPARK WES public dataset, including 44,304 ASD cases, 14,368 siblings, 17,332 fathers, and 29,724 mothers. Representative images for rare variant analysis from 263 Chinese Han autism patients and 294 healthy controls, as well as data from the SFARI SPARK WES public dataset, including 44,304 ASD cases, 14,368 siblings, 17,332 fathers, and 29,724 mothers. Statistical analysis was performed using the one-sided Mann-Whitney U test and the one-sided Fisher’s exact test, with correction applied using the Benjamini-Hochberg (B-H) method. *P < 0.05, **P < 0.01, ***P < 0.001, n. s. not significant.

To target the rare variants in key molecules of the IGF signaling pathway, we analyzed whole-exome sequencing data on a case-control cohort consisting of 263 Chinese Han individuals with autism and 294 healthy controls (Figure 2A; Table 1). The results indicated that approximately 25% of autistic patients carried rare variants in key molecules of the IGF signaling pathway, with the significantly higher proportion when compared to healthy controls (Figure 2B), suggesting that the IGF signaling pathway might play a critical role in the pathogenesis of Chinese Han autistic patients.

**TABLE 1:** Key molecules of the IGF signaling pathway.

Ensembl ID	Official symbol	Subcellular localization
ENSG00000017427	*IGF1*	Extracellular matrix
ENSG00000167244	*IGF2*	Extracellular matrix
ENSG00000140443	*IGF1R*	Cytomembrane
ENSG00000197081	*IGF2R*	Cytomembrane
ENSG00000146678	*IGFBP1*	Extracellular matrix
ENSG00000115457	*IGFBP2*	Extracellular matrix
ENSG00000146674	*IGFBP3*	Extracellular matrix
ENSG00000141753	*IGFBP4*	Extracellular matrix
ENSG00000115461	*IGFBP5*	Extracellular matrix
ENSG00000167779	*IGFBP6*	Extracellular matrix
ENSG00000142208	*AKT1*	Cytosol
ENSG00000145675	*PIK3R1*	Cytosol
ENSG00000102882	*MAPK3*	Cytosol
ENSG00000100030	*MAPK1*	Cytosol
ENSG00000169032	*MAP2K1*	Cytosol
ENSG00000126934	*MAP2K2*	Cytosol
ENSG00000108443	*RPS6KB1*	Cytosol
ENSG00000187840	*EIF4EBP1*	Cytosol

A total of 72 rare variants in key molecules of the IGF signaling pathway were identified among Chinese Han individuals with autism and control, of which 26 (approximately 36%) were predicted to be deleterious. These variants were located in 10 key molecules of the IGF signaling pathway (Figure 2C). Based on their functions and categories, these key molecules were classified into IGFBPs, IGFs, IGF-Rs, PI3K/AKT signaling pathway factors, and MAPK signaling pathway factors, accounting for 11.5%, 7.7%, 50%, 15.4%, and 15.3% of the total rare deleterious variants, respectively. Notably, rare deleterious variants in IGF receptor genes were significantly more frequent compared to other molecules (Figure 2D). Subsequently, we filtered for rare deleterious variants present exclusively in ASD cases. Our analysis identified such variants in *IGF1R*, *IGFBP5*, *AKT1*, *MAP2K1*, *MAP2K2*, and *RPS6KB1* (Table 2).

**TABLE 2:** Rare deleterious variants in key molecules of the IGF signaling pathway which present exclusively in ASD cases.

	Chr	Gene	Location	Ref	Alt	Predict domain	Numbers of sample found in Chinese Han ASD patients	Numbers of sample found in SFARI SPARK ASD patients
1	chr15	IGF1R	99434814	G	A	Furin-like	1	
2	chr15	IGF1R	98707687	C	T	LCR		2
3	chr15	IGF1R	98708078	G	A	FU		1
4	chr15	IGF1R	98891579	C	T	FU		1
5	chr15	IGF1R	98911351	C	T	FN3		1
6	chr15	IGF1R	98913054	A	C	FN3		1
7	chr15	IGF1R	98934854	G	A			1
8	chr15	IGF1R	98939339	G	T	TyrKc		1
9	chr15	IGF1R	98948659	G	A	TyrKc		1
10	chr15	IGF1R	98957383	A	G			1
11	chr2	IGFBP5	217541592	C	T	Thyroglobulin_1	1	
12	chr14	AKT1	105239823	G	A	STKc_PKB_alpha	1	
13	chr14	AKT1	105241519	T	C	STKc_PKB_alpha	1	
14	chr15	MAP2K1	66727430	G	A		1	
15	chr17	RPS6KB1	57990136	C	T	S_TKc	2	
16	chr19	MAP2K2	4102403	C	T	PKc_MEK2	1	

Given the pivotal role of IGF1R in the IGF signaling pathway and the higher frequency of rare deleterious variants in IGF receptors, we expanded our sample size using the SFARI SPARK public WES database, which includes 44,304 ASD patients, 14,368 siblings, 17,332 fathers, and 29,724 mothers, to focused on *IGF1R* and further validate our findings. The results revealed a significantly higher number of rare *IGF1R* variants in ASD patients compared to healthy siblings (Figure 2E). Additionally, we identified 9 rare deleterious variants of *IGF1R* that were exclusively present in ASD patients (Figure 2F; Table 2). These findings suggesting that IGF1R may play a critical role in the pathogenesis of autism and warrant further analysis and investigation.

### 3.2 Structural and functional implications of IGF1R mutations in ASD

**FIGURE 3:**
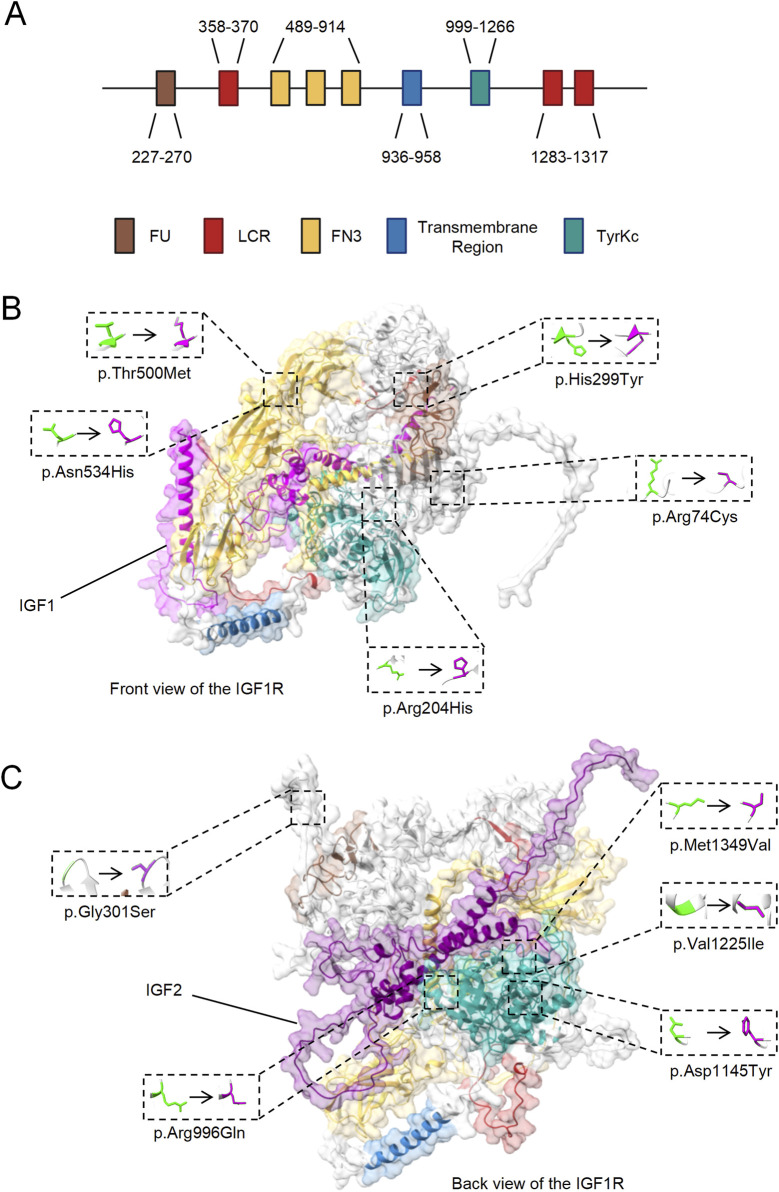
Localization of Rare Deleterious Mutations in IGF1R Domains. **(A)** Schematic representation of IGF1R domains, highlighting the possible domin location of Furin-like repeats (FU), Low Complexity Region (LCR), Fibronectin type III (FN3), Transmembrane Region, and Tyrosine kinase catalytic (TyrKc) predicted by SMART. **(B)** Front view of structural model of IGF1R in complex with IGF1, showing the locations of the mutations exclusively present in ASD patients. **(C)** Back view of structural model of IGF1R in complex with IGF2, showing the locations of the mutations exclusively present in ASD patients.

The rare deleterious mutations were found in the Furin-like repeats (FU) domain, Fibronectin type III (FN3) domain, and Tyrosine kinase catalytic (TyrKc) domain of IGF1R (Figures 3A–C; Table 2). The FU and FN3 domains, located extracellularly, are essential for ligand binding and receptor dimerization. Mutations here might impair these functions and reducing the signaling efficiency. The intracellular TyrKc domain is crucial for tyrosine kinase activity, initiates key signaling including the PI3K/Akt and MAPK pathways. Mutations in this domain can severely disrupt signal transduction, potentially leading to abnormal cell proliferation, differentiation, migration and metabolism.

These results suggest that structural and functional abnormalities in IGF1R might contribute to neurodevelopmental anomalies, potentially leading to autism.

### 3.3 High expression of IGF1R and IGF2R during human cortical development and their potential role in ASD

**FIGURE 4:**
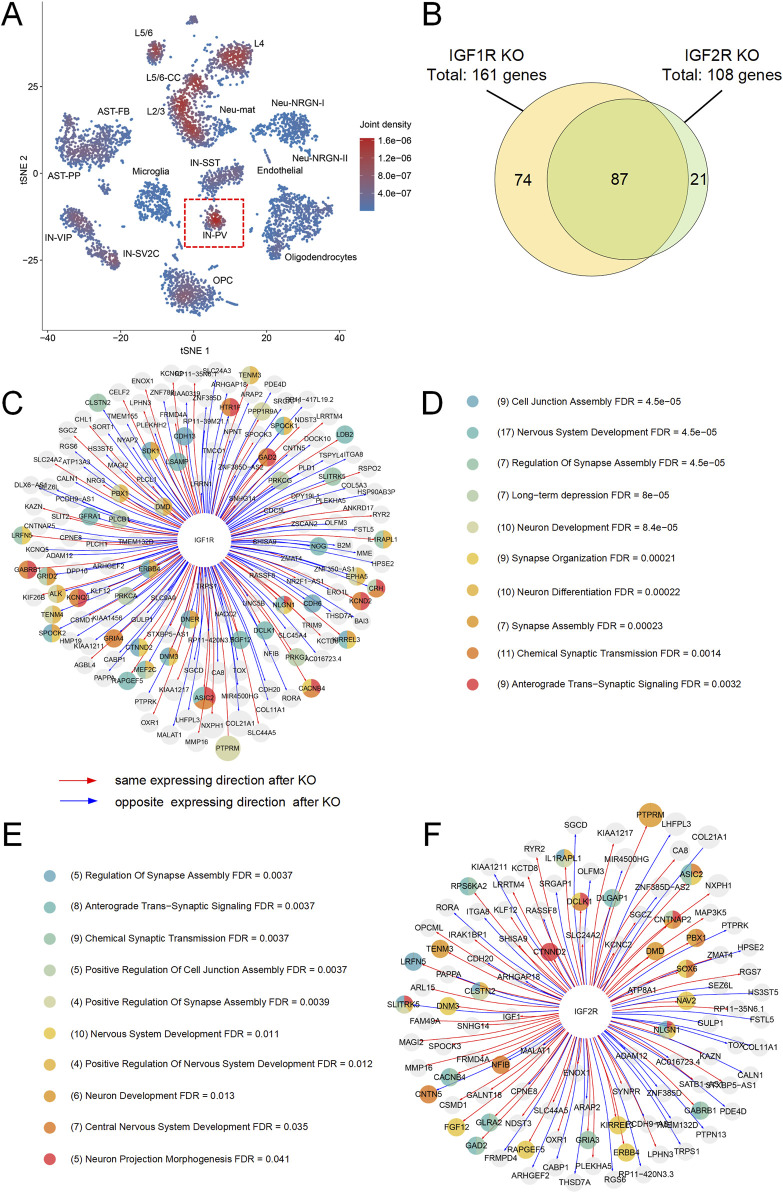
Analysis of *IGF1R* and *IGF2R* Expression and Impact on IN-PV Neurons. **(A)** t-SNE plot showing the expression overlap of *IGF1R* and *IGF2R* in IN-PV neurons. The joint domain expression is highlighted in red. **(B)** Venn diagram illustrating the overlap of differentially expressed genes following virtual knockout (KO) of *IGF1R* and *IGF2R*. A total of 161 genes were perturbed by *IGF1R* KO, and 108 genes were perturbed by *IGF2R* KO, with 87 genes overlapping between the two conditions. **(C)** Network of differentially expressed genes after *IGF1R* KO, with nodes colored by functional categories. **(D)** Enrichment categories for differentially expressed genes following *IGF1R* KO, highlighting processes such as cell junction assembly, synaptic transmission, and neurodevelopment. **(E)** Enrichment categories for differentially expressed genes following *IGF2R* KO, emphasizing processes related to synaptic function, neuronal projection morphology, and neurodevelopment. **(F)** Network of differentially expressed genes after *IGF2R* KO, with nodes colored by functional categories.

During human embryonic development from 8 to 17 weeks, both *IGF1R* and *IGF2R* are highly expressed in the cerebral cortex (Supplementary Figure S1A), suggesting that these receptors might play a critical role in cortical development and function. Single-cell transcriptomic analysis of postmortem cortical tissue from children aged 4–7 years revealed high expression levels of *IGF1R* and *IGF2R* in L2/3, L4, L5/6, L5/6-CC, IN-VIP, IN-SV2C, IN-SST, and IN-PV neurons (Supplementary Figures S1B–D). Co-localization analysis revealed a significant overlap in the expression of *IGF1R* and *IGF2R* within L2/3, L4, L5/6, L5/6-CC, and IN-PV neurons (Figure 4A). These findings indicate that these neuronal populations may be particularly sensitive to IGF signaling pathways, which could be critical for their development and functional regulation during early childhood. Previous studies have shown an imbalance in the excitation/inhibition (E/I) ratio in the brains of individuals with autism, which might be associated with dysfunctional IN-PV neurons (Luo et al., 2024; Pagano et al., 2023; Lee et al., 2021). These findings suggest that the role of IN-PV neurons in ASD may be attributable to aberrant function of IGF receptors. Consequently, our subsequent analyses focused specifically on this neuronal subtype.

To identify the effect of *IGF1R* and *IGF2R* on IN-PV cells, we extract the scRNA-seq data of cortex IN-PV cells and used the expression matrix of 36,501 genes × 72 cells from 4 to 7 years old children postmortem sample as the input for scTenifoldKnk. The final results of scTenifoldKnk analysis contained 161 virtual KO perturbed genes for *IGF1R* and 108 virtual KO perturbed genes for *IGF2R* (FDR < 0.05, Figure 4B). The enrich analysis showed that after the virtual knockout of *IGF1R* and *IGF2R*, differentially expressed genes were predominantly enriched in processes associated with neurodevelopment, neuronal projection morphology, and synaptic function (Figures 4C–F). Notably, approximately 80% of the differentially expressed genes following *IGF2R* knockout overlapped with those observed after *IGF1R* knockout (Figure 4B). Among these, 18 genes are known autism susceptibility genes, which were mainly enriched in processes associated with nervous system development (Figures 4C–F; Table 3). These findings suggest that both receptors may play significant roles in neurodevelopment of IN-PV neurons and the pathogenesis of autism. Furthermore, the impact of IGF2R on these processes might be mediated through IGF1R. Additionally, IGF1R is likely a critical factor in the development and maintenance of IN-PV neurons.

**TABLE 3:** Differentially expressed autism susceptibility genes after knock out *IGF1R* and *IGF2R* in IN-PV cells.

Gene	Distance	FC	p.Value	p.Adj	Ensembl.ID	Chromosome	Gene.Score (Safri)	Syndromic	Number of Reports
*ARHGEF2*	5.65E-08	1.54E+01	8.49E-05	1.00E-02	ENSG00000116584	1	3	0	7
*CNTN5*	8.69E-08	3.65E+01	1.49E-09	3.49E-07	ENSG00000149972	11	2	0	11
*CSMD1*	8.19E-08	3.25E+01	1.19E-08	2.51E-06	ENSG00000183117	8	2	0	19
*CTNND2*	8.53E-08	3.52E+01	2.96E-09	6.49E-07	ENSG00000169862	5	2	0	15
*DMD*	6.58E-08	2.09E+01	4.72E-06	7.46E-04	ENSG00000198947	X	S	1	46
*HS3ST5*	5.19E-08	1.30E+01	3.06E-04	3.17E-02	ENSG00000249853	6	2	0	8
*IL1RAPL1*	5.83E-08	1.65E+01	4.94E-05	6.39E-03	ENSG00000169306	X	2	0	27
*ITGA8*	6.48E-08	2.03E+01	6.48E-06	9.81E-04	ENSG00000077943	10	3	0	7
*KCNC2*	6.07E-08	1.79E+01	2.37E-05	3.27E-03	ENSG00000166006	12	3	0	13
*KIRREL3*	1.53E-07	1.14E+02	1.32E-26	1.42E-23	ENSG00000149571	11	2	0	19
*LRFN5*	7.54E-08	2.75E+01	1.57E-07	2.81E-05	ENSG00000165379	14	2	0	7
*NFIB*	8.87E-08	3.81E+01	6.59E-10	1.65E-07	ENSG00000147862	9	2	1	7
*NLGN1*	1.08E-07	5.60E+01	7.09E-14	2.82E-11	ENSG00000169760	3	2	0	21
*NXPH1*	2.26E-07	2.47E+02	1.10E-55	2.96E-52	ENSG00000122584	7	2	0	6
*PBX1*	1.01E-07	4.92E+01	2.28E-12	7.90E-10	ENSG00000185630	1	2	0	8
*RORA*	9.68E-08	4.54E+01	1.62E-11	5.29E-09	ENSG00000069667	15	S	1	25
*SLC24A2*	9.66E-08	4.52E+01	1.78E-11	5.61E-09	ENSG00000155886	9	2	0	4
*SLITRK5*	5.98E-08	1.73E+01	3.16E-05	4.19E-03	ENSG00000165300	13	2	0	10

### 3.4 Co-expression patterns of IGF1R in brain organoids of autism patients

**FIGURE 5:**
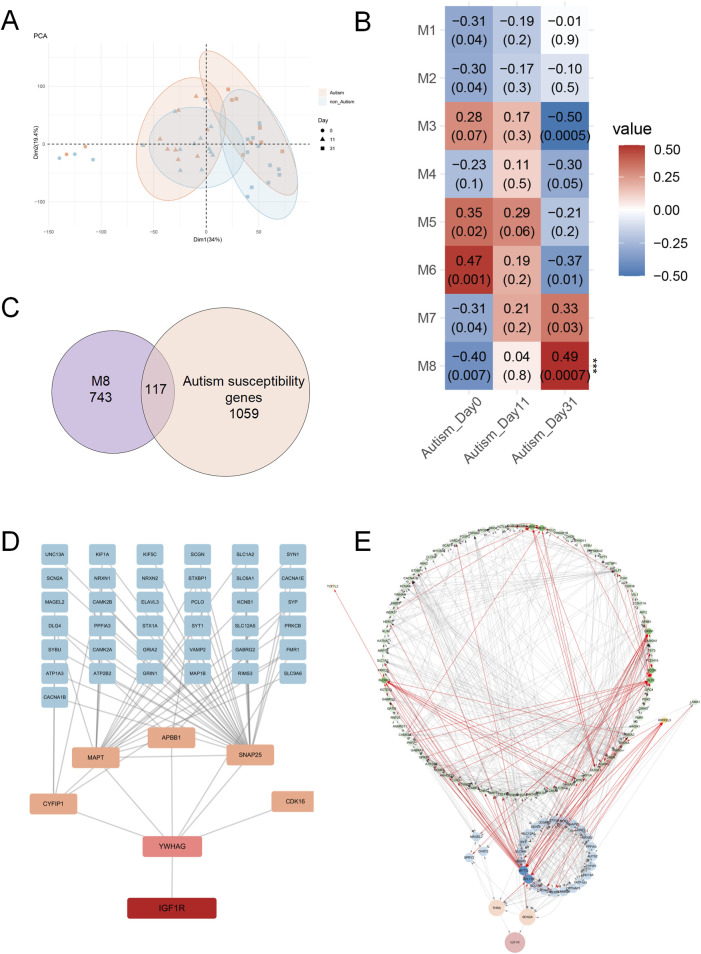
Analysis of *IGF1R* in Brain Organoids Derived from ASD Patients and Healthy Controls. **(A)** Principal Component Analysis (PCA) of transcriptome sequencing data (GSE61476) from brain organoids at three developmental stages (day 0, day 11, and day 31). Significant differences in gene expression patterns between ASD and control groups were observed at days 11 and 31, but not at day 0. **(B)** Heatmap of module-trait relationships from Weighted Gene Co-Expression Network Analysis (WGCNA) of brain organoid genes at three time points. Module 8, containing *IGF1R*, showed a significant association with the autistic group on day 31. **(C)** Venn diagram showing the overlap between genes in module 8 and known autism susceptibility genes. A total of 117 genes overlapped. **(D)** Protein-protein interaction network analysis indicating that IGF1R interacts with the protein of ASD susceptibility gene YWHAG, mediating interactions with other ASD susceptibility genes’ protein such as CYFIP1, MAPT, APBB1, SNAP25, and CDK16. **(E)** Regulatory network analysis of module 8, illustrating that *IGF1R* is directly regulated by ASD susceptibility genes *THRA* and *SCN2A*. Genes highlighted in the network are those significantly differentially expressed in autism brain organoids. Connections originating from or pointing to these genes are marked in red.

To further investigate the role of IGF1R in autism, this study utilized transcriptome sequencing data (GSE61476) from brain organoids induced from stem cells derived from patients with ASD and healthy controls. Principal Component Analysis (PCA) revealed significant changes in gene expression patterns between the autism and control groups as development progressed. At day 0 of culture, there were no significant differences in gene expression patterns between the ASD and controls. However, at days 11 and 31, the autistic group exhibited significantly different expression patterns compared to the control (Figure 5A). These results suggest that brain organoids in the autistic group might undergo distinct gene regulatory processes during early developmental stages, leading to differences in expression patterns at later stages, particularly at days 11 and 31.

We then performed Weighted Gene Co-Expression Network Analysis (WGCNA) on brain organoid genes from autism and control groups at three time points (Figure 5B). We found that module 8, containing *IGF1R*, showed significant association with the autistic group on day 31 (Figure 5B). The genes in this modules partially overlapped with known ASD susceptibility genes (Figure 5C), suggesting that the IGF1R plays a time-dependent and stage-specific role in the pathogenesis of ASD, potentially acting through distinct gene networks at different developmental stages. *IGF1R* and its co-expressed ASD susceptibility genes, showed GO enrichment results indicating that these genes might affect synaptic function and neuronal communication during neurodevelopment by regulating the function of ion channels at the synapse (Supplementary Figures S2A–C).

The protein-protein interaction analysis revealed that IGF1R initially interacts with the protein of ASD susceptibility gene YWHAG, thereby mediating its interactions with CYFIP1, MAPT, APBB1, SNAP25, CDK16, and other ASD susceptibility genes’ protein (Figure 5D). YWHAG mediates signal transduction by binding to phosphoserine-containing proteins and interacts with RAF1 and protein kinase C. Both ablation and overexpression of YWHAG delay neuronal migration in the developing cerebral cortex (Wachi et al., 2016; Cornell et al., 2016). These results indicate that structural and function abnormalities in the TyrKc domain of IGF1R may impair its interaction with YWHAG, potentially leading to neurodevelopmental abnormalities and autism.

Regulatory network analysis of module 8 indicated that *IGF1R* is directly regulated by ASD susceptibility genes *THRA*, which is a nuclear hormone receptor for triiodothyronine, and *SCN2A*, which is a member of the sodium channel alpha subunit gene family (Figure 5E). Several researches show that they are strongly associated with ASD (An et al., 2018; Feliciano et al., 2019; Kalikiri et al., 2017; Ruzzo et al., 2019; Adney et al., 2020; Iossifov et al., 2012; Sanders et al., 2012; Tavassoli et al., 2014; Wang et al., 2020). These findings suggest that IGF1R may play a critical role in the pathophysiology of ASD through its regulation by *THRA* and *SCN2A*, implicating a potential mechanistic link between thyroid hormone signaling, sodium channel function, and autism spectrum disorder.

**FIGURE 6:**
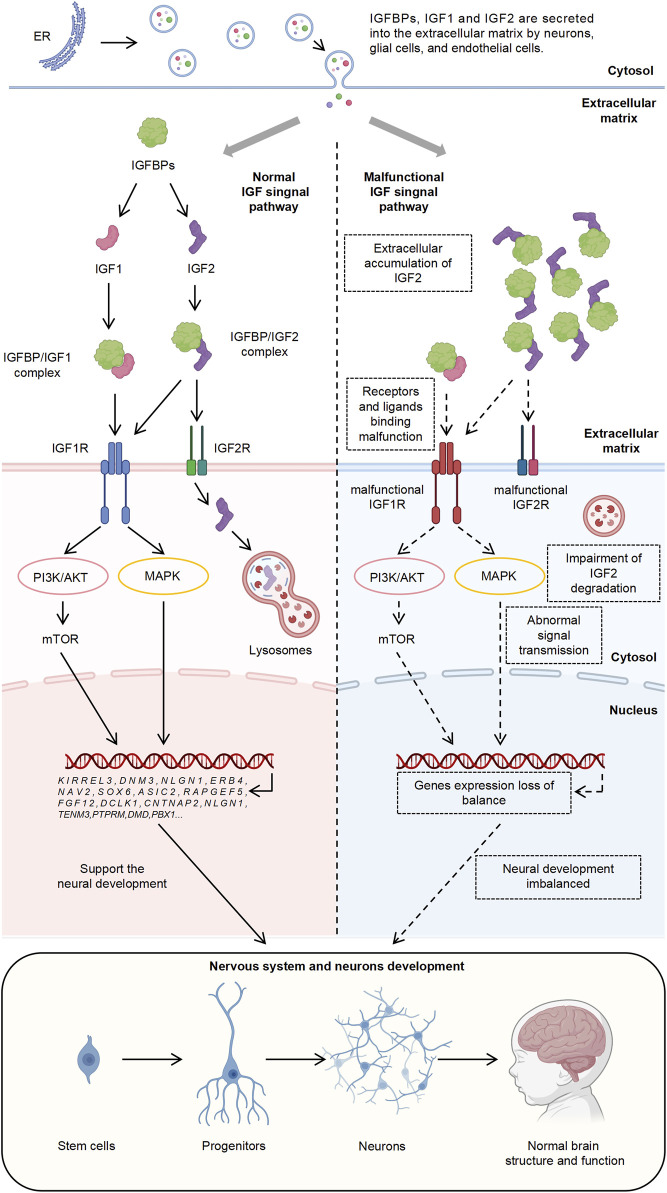
Mechanistic Illustration of IGF Signaling Pathway and Its Role in ASD Pathogenesis. The diagram depicts the normal and dysfunctional IGF signaling pathways and integrates findings from this study.

Overall, these results underscore the significant role of IGF1R in the pathogenesis of ASD and highlight the complex function of IGF1R within the IGF signaling pathway. This complexity involves multiple intermediary molecules or regulatory factors and multi-layered regulatory mechanisms that influence the pathological processes of ASD (Figure 6).

## 4 Discussion

The IGF signaling pathway plays a crucial role in cell growth, development, and metabolism (Takahashi, 2019). Recent studies suggest that IGF1 analogs or IGF2 could help alleviate social and behavioral deficits in autism (Neul et al., 2022; Pizzarelli et al., 2023), indicating a potential link between IGF signaling disruptions and autism. Furthermore, the effects of IGF1 and IGF2 on cells are contingent upon the downstream signaling pathways activated following their binding to IGF1R (Andersson et al., 2019; Barclay et al., 2019). In this study, we analyzed whole-exome sequencing data from two different datasets to investigate rare genetic variants in the IGF signaling pathway. Our findings revealed that the number of rare *IGF1R* variants in ASD patients compared to healthy controls were significantly increased. The rare deleterious mutations were mainly found in the Furin-like repeats (FU) domain, Fibronectin type III (FN3) domain, and Tyrosine kinase catalytic (TyrKc) domain of IGF1R. Mutations in this domain can severely disrupt signal transduction, potentially leading to abnormal cell proliferation, differentiation, migration and metabolism (Altieri et al., 2019; Barclay et al., 2019; Bhalla et al., 2022; Boguszewski and Boguszewski, 2019; Crudden et al., 2019). These findings suggesting that IGF1R may play a critical role in the pathogenesis of autism.

The significant expression of *IGF1R* and *IGF2R* in the human cerebral cortex from the 8th to the 17th week during embryonic development strongly suggests their crucial role in cortical development. Our detailed single-cell analysis further revealed that *IGF1R* and *IGF2R* are predominantly expressed in neurons, with markedly lower expression levels observed in glial cells. This differential expression pattern underscores the importance of these receptors in neuronal development and functional maintenance. Particularly noteworthy is the co-localization of *IGF1R* and *IGF2R* in IN-PV neurons, a subset of inhibitory neurons that play a critical role in maintaining the excitatory-inhibitory balance within the nervous system by modulating neuronal excitability. Previous research has established a link between abnormalities in IN-PV neurons and disruptions in excitatory-inhibitory balance in individuals with autism (Luo et al., 2024; Pagano et al., 2023; Lee et al., 2021), suggesting that these cellular anomalies may contribute to the clinical manifestations of the disorder. Our findings align with these earlier observations, providing further evidence of the involvement of IGF1R and IGF2R in this context.

In-depth analysis revealed that IGF1R and IGF2R regulate a group of 182 genes within IN-PV neurons, including 18 genes known to be associated with autism. Functional enrichment analysis indicated that these genes are significantly involved in biological processes related to neuronal development, nervous system development, and synaptic morphology and function. This suggests that disruptions in the structure and function of IGF1R and IGF2R could lead to developmental and functional impairments in IN-PV neurons, potentially contributing to the pathophysiology of autism. An intriguing aspect of our findings is that approximately 80% of the differentially expressed genes following *IGF2R* knockout overlap with those affected by *IGF1R* knockout. This substantial overlap implies that the effects of IGF2R on IN-PV neurons might be mediated through IGF1R signaling pathways. This insight opens up new avenues for understanding the molecular mechanisms underlying IN-PV neurons function and their role in neurodevelopmental disorders such as autism.

Weighted gene co-expression network analysis of brain organoids derived from autism patients revealed that module 5 (Figure 5B), containing key molecules of the *IGF1*, was associated with the autism group on day 11, while module 8, containing *IGF1R*, was significantly associated with the autism group on day 31. *IGF1*, located in module 5, and other autism susceptibility genes in the same module were subjected to GO enrichment analysis, revealing that these co-expressed genes might influence neural development in early stages by regulating processes such as DNA binding, histone binding, and RNA splicing within the nucleus (Supplementary Figures S4A–C). Conversely, module 8, which includes *IGF1R* and its co-expressed autism susceptibility genes, showed GO enrichment results indicating that these genes might influence synaptic function and neuronal communication in the neural development by regulating various ion channel functions at the synapse (Supplementary Figures S2A–C). These modules shared genes with known autism susceptibility genes but did not overlap with each other (Supplementary Figures S3A), suggesting that the IGF signaling pathway may have time-dependent and stage-specific roles in the pathogenesis of autism, potentially acting through distinct gene networks at different developmental stages.

Further insights were gained through protein-protein interaction analysis, which revealed that IGF1R initially interacts with the protein of autism susceptibility gene YWHAG. This interaction appears to mediate subsequent interactions with a network of other autism-related genes’ protein, including CYFIP1, MAPT, APBB1, SNAP25, and CDK16. Regulatory network analysis of module 8 indicated that *IGF1R* is directly regulated by autism susceptibility genes *THRA* and *SCN2A*. These results underscore the pivotal role of IGF1R in the pathogenesis of autism and highlight the intricate and multifaceted function of IGF1R within the IGF signaling pathway. This complexity is characterized by the involvement of multiple intermediary molecules or regulatory factors and multi-layered regulatory mechanisms that modulate the pathological processes associated with autism. Notably, recent studies have demonstrated that the conditional knockout of the *GIGYF*1 gene in mice disrupts IGF1R/ERK signaling pathways, resulting in autism-like behaviors (Chen et al., 2022), further supporting our findings. And our findings suggest that targeting the IGF1R could offer new therapeutic strategies for autism, emphasizing the need for further research to unravel the precise molecular mechanisms and interactions at play.

On the other hand, although *IGF1* is considered an autism susceptibility gene, its disease risk score in the SFARI database remains relatively low due to limited research on its role in autism pathogenesis. Recent phase III clinical studies have shown that IGF1 analogs can improve autism-like behaviors in patients with Rett syndrome (Neul et al., 2023). These findings suggest two important implications: firstly, there is a need to re-evaluate the role of IGF1 in autism despite its lower genetic ranking; secondly, targeting the IGF signaling pathway could be explored as a potential therapeutic strategy for autism-related symptoms.

Our whole-exome sequencing identified rare deleterious variants of *IGF1* (Figure 2C). Single-cell transcriptomic analysis of postmortem brain cortex from ASD children indicated that *IGF1* is highly expressed only in interneurons, particularly in IN-PV neurons (Supplementary Figures S3C, D). Differential expression analysis following *IGF1* knockout in IN-PV neurons revealed enrichment of genes involved in neuronal development (Supplementary Figures S3E, F). Additionally, transcriptomic analysis of brain organoids derived from autism patients demonstrated a significant downregulation of *IGF1* on day 11 of *in vitro* culture (Supplementary Figure S3B). Protein interaction analysis showed that IGF1 forms a cluster with proteins involved in synaptic growth, influencing functions such as protein serine/threonine kinase activity within glutamatergic synapses and axonal growth cones (Supplementary Figures S5A–D). Regulatory inference using the Genie3 algorithm identified direct regulation of *IGF1* by *SIK1*, *MFRP*, *CHD7*, *NIPBL*, *EXOC5*, and *ARID2*, with *SIK1* and *SPARCL1* significantly downregulated in autism brain organoids (Supplementary Figure S5E), potentially affecting IGF1 expression.

Furthermore, previous studies have demonstrated that the functional effects of IGF1 are mediated through IGF1R, which, upon binding with IGF1, activates downstream PI3K/AKT and MAPK signaling pathways, thereby regulating cellular functions (Forbes et al., 2020; Takahashi, 2019). This underscores the significance of the IGF signaling pathway in neurodevelopment and the pathogenesis of autism, as well as the central role of IGF1R within the entire IGF signaling cascade (Figure 6).

Previous research on the IGF signaling pathway has primarily concentrated on tumors and cancers, with limited studies addressing its impact on neurodevelopment (Altieri et al., 2019; Kamei, 2020; Hua et al., 2019; Manzella et al., 2019). Autism is profoundly influenced by genetic factors, yet the genetic heterogeneity associated with autism is remarkably high (Iakoucheva et al., 2019; Vicari et al., 2019; Joon et al., 2021). Over recent years, numerous autism susceptibility genes have been identified and validated; however, the pathogenic pathways through which these genes exert their effects remain diverse and lack a unified pathological mechanism (Al-Dewik and Alsharshani, 2020; Bhandari et al., 2020; Yasuda et al., 2023). Utilizing a comprehensive multi-omics approach, we integrated whole-exome sequencing of ASD patients, single-cell transcriptomic analysis of postmortem cortical tissue from autism children aged 4–7 years, transcriptomic analysis of autism brain organoids, weighted gene co-expression network analysis, protein-protein interaction network analysis, and gene regulatory network analysis to elucidate potential common pathogenic mechanisms involving autism susceptibility genes by linking key molecules within the IGF signaling pathway. Our findings underscore the central role of IGF1R as a pivotal node within this pathway, acting as a crucial intermediary that bridges upstream regulatory signals and downstream effectors. These findings suggest that targeting the IGF signaling pathway could represent a promising therapeutic strategy for autism.

In this study, we focused primarily on rare mutations, which has facilitated the elucidation of the critical roles these mutations play in the onset and progression of the disease. However, this approach has certain limitations. Specifically, it may overlook the contributions of common variants. In reality, common variants may also play significant roles in disease development and progression. Therefore, future research should place greater emphasis on the study of common variants to provide a more comprehensive understanding of the relationship between genetic variations and disease. Additionally, our current research is predominantly centered on genetic data analysis, with relatively insufficient exploration of the pathogenic molecular mechanisms and behavioral aspects following gene deletion. Future studies using induced pluripotent stem cell technology to cultivate brain organoids, as well as gene editing techniques in animal models to manipulate *IGF1R* and related genes, could further elucidate the mechanisms of the IGF signaling pathway in the onset of autism.

In conclusion, our study underscores the importance of IGF1R within the IGF signaling pathway and its potential role in the pathogenesis of autism. By integrating multiple layers of genomic and transcriptomic data, we have identified a more convergent pathological pathway that may account for the genetic heterogeneity observed in autism. Future research should aim to validate these findings and explore the therapeutic potential of modulating the IGF signaling pathway in autism.

## Data Availability

The datasets presented in this study can be found in online repositories. The names of the repository/repositories and accession number(s) can be found in the article/Supplementary Material.
