# Supplementary material for: Unveiling the role of IGF1R in autism spectrum disorder: a multi-omics approach to decipher common pathogenic mechanisms in the IGF signaling pathway
Source: Front Genet. 2024 Sep 23;15:1483574. doi: 10.3389/fgene.2024.1483574 (PMC11456441; doi:10.3389/fgene.2024.1483574)
Supplement: Supplementary file 1 [file DataSheet1.PDF]

Supplementary Material

Supplementary Figures

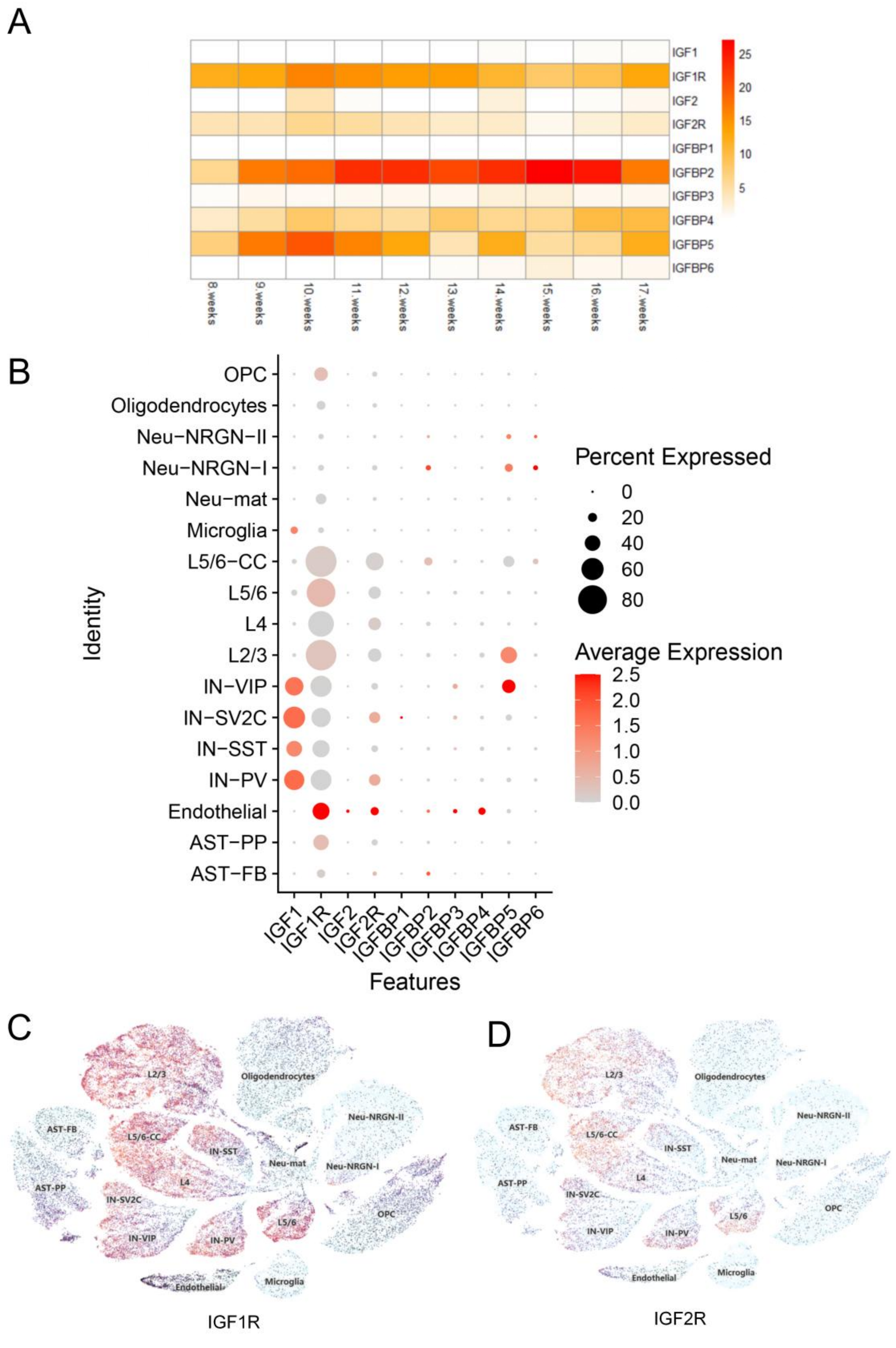

Supplementary Figure 1. Expression of *IGF1R* and *IGF2R* During Human Embryonic Development and in Postmortem Cortical Tissue

A. Heatmap illustrating the high expression levels of *IGF1R* and *IGF2R* in the cerebral cortex during human embryonic development from 8 to 17 weeks. Data from EMBL-EBI database(E-MTAB-4840).
B. Single-cell transcriptomic analysis of postmortem cortical tissue from children aged 4-7 years, showing high expression levels of *IGF1R* and *IGF2R* in various neuronal subtypes, including L2/3, L4, L5/6, L5/6-CC, IN-VIP, IN-SV2C, IN-SST, and IN-PV neurons. The size of the dots represents the percentage of cells expressing the gene, while the color intensity indicates the average expression level.
C. Spatial distribution of *IGF1R* expression in the postmortem cortical tissue.
D. Spatial distribution of *IGF2R* expression in the postmortem cortical tissue.

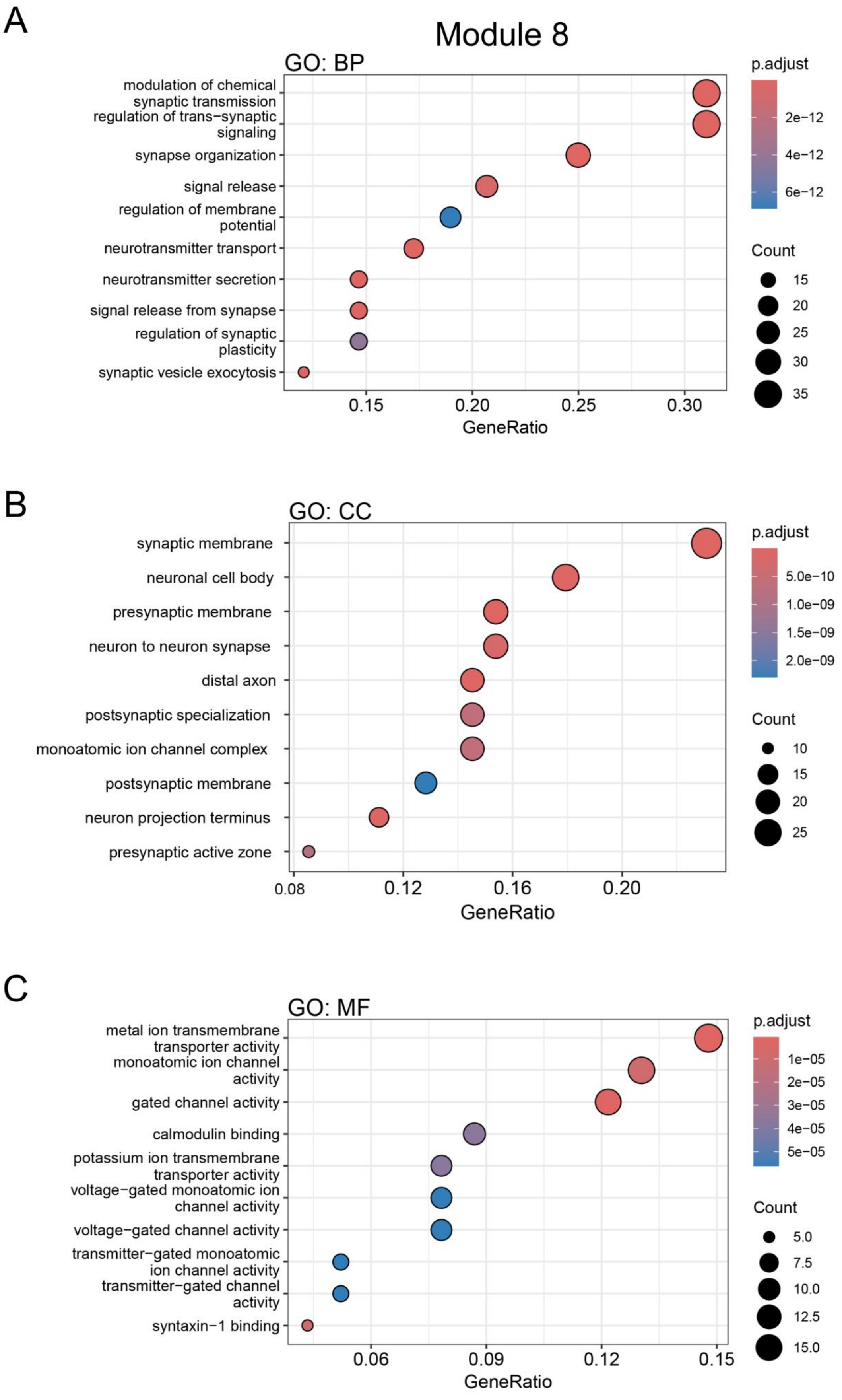
A
Module 8
GO: BP
modulation of chemical synaptic transmission
regulation of trans−synaptic signaling
synapse organization
signal release
regulation of membrane potential
neurotransmitter transport
neurotransmitter secretion
signal release from synapse
regulation of synaptic plasticity
synaptic vesicle exocytosis
0.15
0.20
0.25
0.30
GeneRatio
p.adjust
2e−12
4e−12
6e−12
Count
15
20
25
30
35
B
GO: CC
synaptic membrane
neuronal cell body
presynaptic membrane
neuron to neuron synapse
distal axon
postsynaptic specialization
monoatomic ion channel complex
postsynaptic membrane
neuron projection terminus
presynaptic active zone
0.08
0.12
0.16
0.20
GeneRatio
p.adjust
5.0e−10
1.0e−09
1.5e−09
2.0e−09
Count
10
15
20
25
C
GO: MF
metal ion transmembrane transporter activity
monoatomic ion channel activity
gated channel activity
calmodulin binding
potassium ion transmembrane transporter activity
voltage−gated monoatomic ion channel activity
voltage−gated channel activity
transmitter−gated monoatomic ion channel activity
transmitter−gated channel activity
syntaxin−1 binding
0.06
0.09
0.12
0.15
GeneRatio
p.adjust
1e−05
2e−05
3e−05
4e−05
5e−05
Count
5.0
7.5
10.0
12.5
15.0

Supplementary Figure 2. GO Enrichment Analysis of *IGF1R* and Co-Expressed ASD Susceptibility Genes

A. Gene Ontology (GO) enrichment analysis for biological processes (GO: BP) showing significant enrichment in processes related to synaptic function and neuronal communication, including modulation of chemical synaptic transmission, regulation of neurotransmitter levels, synapse organization, and synaptic vesicle exocytosis.
B. GO enrichment analysis for cellular components (GO: CC) highlighting significant enrichment in components associated with synaptic function, such as the synaptic membrane, presynaptic membrane, postsynaptic membrane, and monoamine ion channel complex.
C. GO enrichment analysis for molecular functions (GO: MF) indicating significant enrichment in functions related to ion channel activity, including metal ion transmembrane transporter activity, monoatomic ion channel activity, and various gated channel activities.

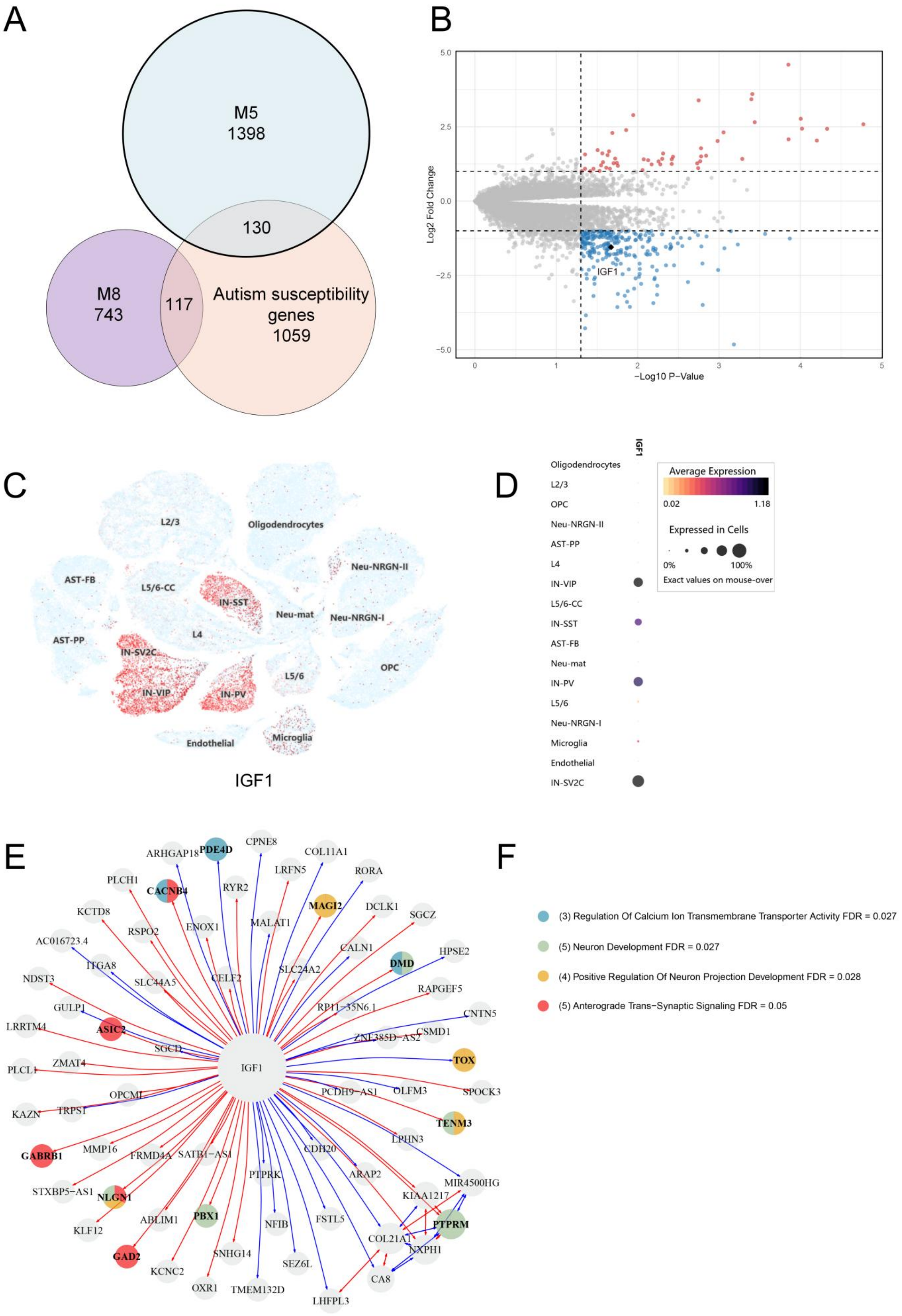
A
M5
1398
130
M8
743
117
Autism susceptibility genes
1059
B
Log2 Fold Change
−Log10 P−Value
IGF1
C
L2/3
Oligodendrocytes
AST-FB
L5/6-CC
IN-SST
Neu-NRGN-II
Neu-mat
Neu-NRGN-I
AST-PP
L4
IN-SV2C
OPC
L5/6
IN-VIP
IN-PV
Endothelial
Microglia
IGF1
D
IGF1
Oligodendrocytes
L2/3
OPC
Neu-NRGN-II
AST-PP
L4
IN-VIP
L5/6-CC
IN-SST
AST-FB
Neu-mat
IN-PV
L5/6
Neu-NRGN-I
Microglia
Endothelial
IN-SV2C
Average Expression
0.02
1.18
Expressed in Cells
0%
100%
Exact values on mouse-over
E
IGF1
F
(3) Regulation Of Calcium Ion Transmembrane Transporter Activity FDR = 0.027
(5) Neuron Development FDR = 0.027
(4) Positive Regulation Of Neuron Projection Development FDR = 0.028
(5) Anterograde Trans−Synaptic Signaling FDR = 0.05

Supplementary Figure 3. Analysis of *IGF1* and Its Role in Autism and Neuronal Development

A. Venn diagram showing the overlap of genes in modules M5 and M8 with known autism susceptibility genes.
B. Transcriptomic analysis of brain organoids derived from autism patients, demonstrating significant downregulation of IGF1 on day 11 of in vitro culture.
C. Single-cell transcriptomic analysis of postmortem brain cortex from children aged 4-7 years, indicating that *IGF1* is highly expressed only in interneurons, particularly in IN-PV neurons.
D. Dot plot showing the average expression levels of *IGF1* in various cell types, highlighting its high expression in IN-PV neurons.
E. Differential expression analysis following *IGF1* knockout in IN-PV neurons, revealing enrichment of genes involved in neuronal development.
F. GO enrichment analysis of differentially expressed genes following *IGF1* knockout, showing significant enrichment in processes related to neuronal development, regulation of calcium ion transmembrane transporter activity, and synaptic signaling.

Module 5

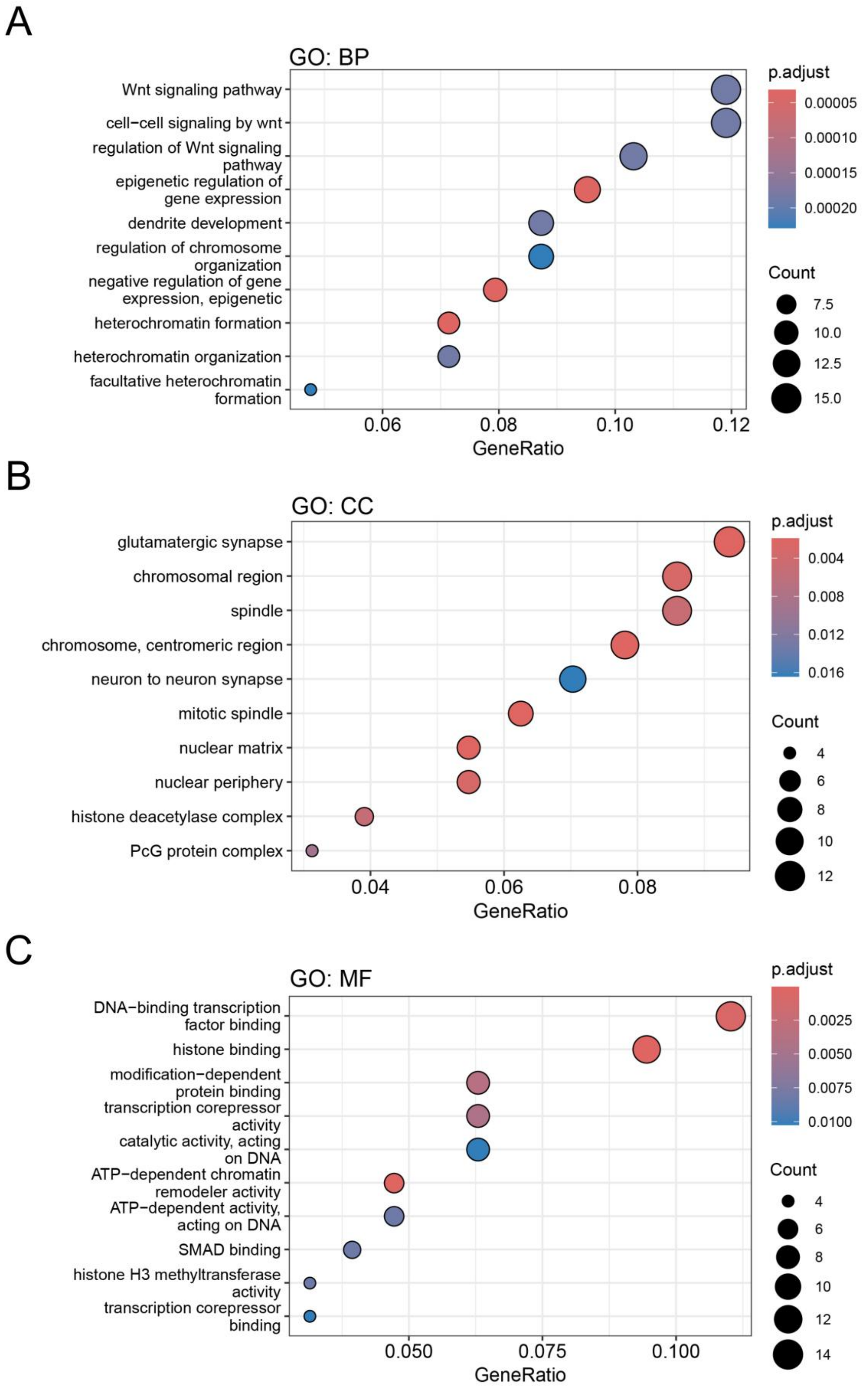

A
GO: BP
Wnt signaling pathway
cell−cell signaling by wnt
regulation of Wnt signaling pathway
epigenetic regulation of gene expression
dendrite development
regulation of chromosome organization
negative regulation of gene expression, epigenetic
heterochromatin formation
heterochromatin organization
facultative heterochromatin formation
0.06
0.08
0.10
0.12
GeneRatio
p.adjust
0.00005
0.00010
0.00015
0.00020
Count
7.5
10.0
12.5
15.0
B
GO: CC
glutamatergic synapse
chromosomal region
spindle
chromosome, centromeric region
neuron to neuron synapse
mitotic spindle
nuclear matrix
nuclear periphery
histone deacetylase complex
PcG protein complex
0.04
0.06
0.08
GeneRatio
p.adjust
0.004
0.008
0.012
0.016
Count
4
6
8
10
12
C
GO: MF
DNA−binding transcription factor binding
histone binding
modification−dependent protein binding
transcription corepressor activity
catalytic activity, acting on DNA
ATP−dependent chromatin remodeler activity
ATP−dependent activity, acting on DNA
SMAD binding
histone H3 methyltransferase activity
transcription corepressor binding
0.050
0.075
0.100
GeneRatio
p.adjust
0.0025
0.0050
0.0075
0.0100
Count
4
6
8
10
12
14

Supplementary Figure 4. GO Enrichment Analysis of Module 5 in Brain Organoids Derived from Autism Patients

A. Gene Ontology (GO) Biological Process (BP) enrichment analysis of genes in module 5, which includes *IGF1* and other autism susceptibility genes. The analysis reveals significant enrichment in processes such as Wnt signaling pathway, cell-cell signaling by Wnt, epigenetic regulation of gene expression, and dendrite development, suggesting a role in neural development during early stages.
B. GO Cellular Component (CC) enrichment analysis of genes in module 5, indicating significant associations with components such as the glutamatergic synapse, chromosomal region, mitotic spindle, nuclear matrix, and histone deacetylase complex, which are crucial for chromosomal organization and synaptic function.
C. GO Molecular Function (MF) enrichment analysis of genes in module 5, highlighting significant enrichment in functions such as DNA-binding transcription factor activity, histone binding, transcription coactivator binding, and ATP-dependent chromatin remodeling, implicating these genes in the regulation of transcription and chromatin structure within the nucleus.

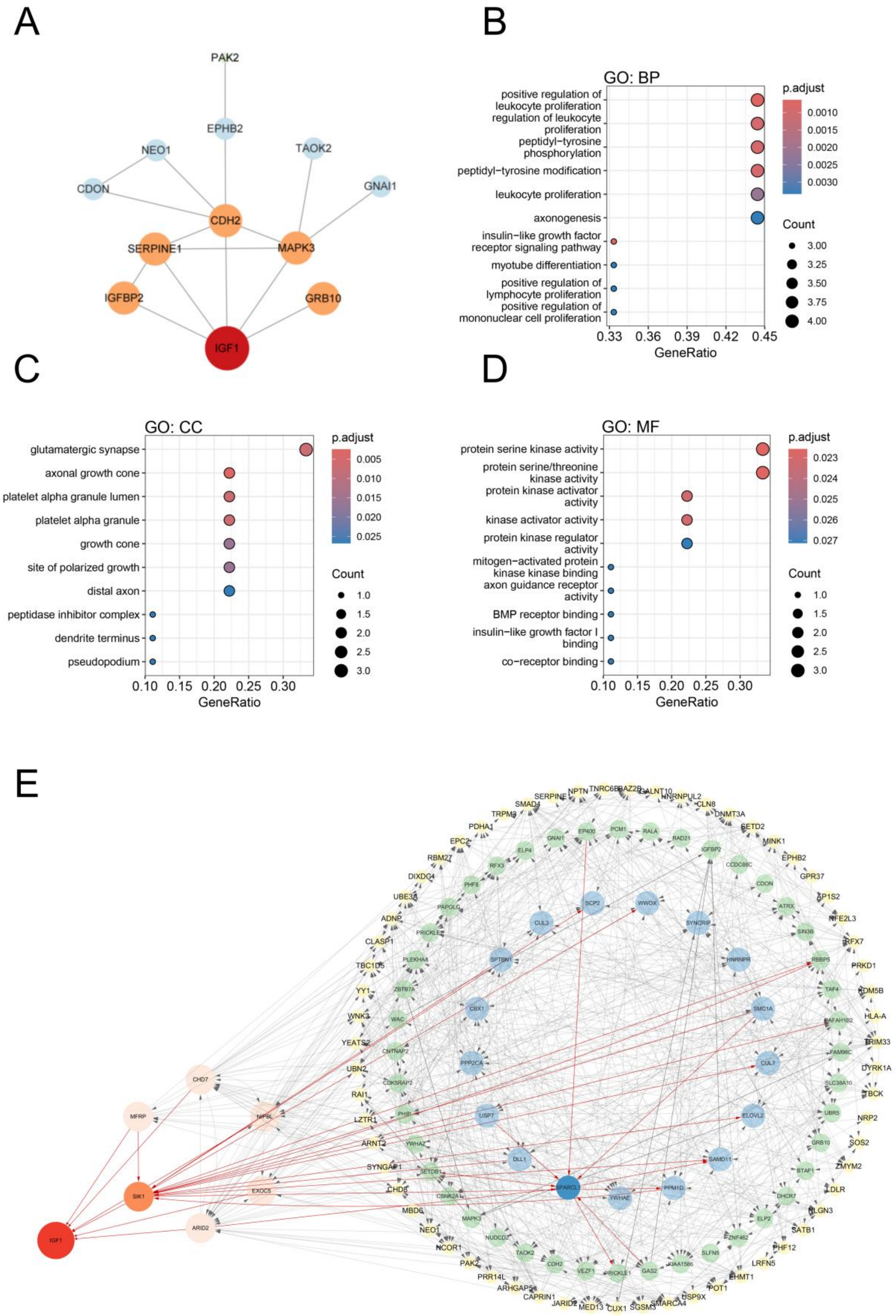
A
PAK2
EPHB2
NEO1
TAOK2
CDON
GNAI1
CDH2
SERPINE1
MAPK3
IGFBP2
GRB10
IGF1
B
GO: BP
positive regulation of leukocyte proliferation
regulation of leukocyte proliferation
peptidyl−tyrosine phosphorylation
peptidyl−tyrosine modification
leukocyte proliferation
axonogenesis
insulin−like growth factor receptor signaling pathway
myotube differentiation
positive regulation of lymphocyte proliferation
positive regulation of mononuclear cell proliferation
0.33 0.36 0.39 0.42 0.45
GeneRatio
p.adjust
0.0010
0.0015
0.0020
0.0025
0.0030
Count
3.00
3.25
3.50
3.75
4.00
C
GO: CC
glutamatergic synapse
axonal growth cone
platelet alpha granule lumen
platelet alpha granule
growth cone
site of polarized growth
distal axon
peptidase inhibitor complex
dendrite terminus
pseudopodium
0.10 0.15 0.20 0.25 0.30
GeneRatio
p.adjust
0.005
0.010
0.015
0.020
0.025
Count
1.0
1.5
2.0
2.5
3.0
D
GO: MF
protein serine kinase activity
protein serine/threonine kinase activity
protein kinase activator activity
kinase activator activity
protein kinase regulator activity
mitogen−activated protein kinase kinase binding
axon guidance receptor activity
BMP receptor binding
insulin−like growth factor I binding
co−receptor binding
0.10 0.15 0.20 0.25 0.30
GeneRatio
p.adjust
0.023
0.024
0.025
0.026
0.027
Count
1.0
1.5
2.0
2.5
3.0
E

Supplementary Figure 5. Protein Interaction and Regulatory Network Analysis of *IGF1* in Brain Organoids Derived from Autism Patients

A. Protein interaction network showing IGF1 clustering with proteins involved in synaptic growth. Key interactions include those influencing protein serine/threonine kinase activity within glutamatergic synapses and axonal growth cones.
B. Gene Ontology (GO) Biological Process (BP) enrichment analysis of proteins interacting with IGF1, highlighting significant processes such as positive regulation of synapse assembly, peptidyl-tyrosine phosphorylation, and leukocyte proliferation, which are critical for synaptic development and immune responses.
C. GO Cellular Component (CC) enrichment analysis of proteins interacting with IGF1, indicating significant associations with components such as the glutamatergic synapse, axonal growth cone, and postsynaptic density, which are essential for synaptic signaling and axonal guidance.
D. GO Molecular Function (MF) enrichment analysis of proteins interacting with IGF1, revealing significant enrichment in functions such as protein serine/threonine kinase activity, protein kinase binding, and insulin-like growth factor binding, implicating these proteins in signaling pathways that regulate synaptic and axonal growth.
E. Regulatory network inference using the Genie3 algorithm, identifying direct regulation of *IGF1* by *SIK1*, *MFRP*, *CHD7*, *NIPBL*, *EXOC5*, and *ARID2*. Notably, *SIK1* and *SPARCL1* are significantly downregulated in autism brain organoids, potentially affecting *IGF1* expression and its downstream effects on neural development. Genes highlighted in the network are those significantly differentially expressed in autism brain organoids. Connections originating from or pointing to these genes are marked in red.
